# Microbiome Modulation—Toward a Better Understanding of Plant Microbiome Response to Microbial Inoculants

**DOI:** 10.3389/fmicb.2021.650610

**Published:** 2021-04-08

**Authors:** Gabriele Berg, Peter Kusstatscher, Ahmed Abdelfattah, Tomislav Cernava, Kornelia Smalla

**Affiliations:** ^1^Institute of Environmental Biotechnology, Graz University of Technology, Graz, Austria; ^2^Julius Kühn Institute (JKI) Federal Research Centre for Cultivated Plants, Institute for Epidemiology and Pathogen Diagnostics, Braunschweig, Germany

**Keywords:** holobiont, microbial diversity, healthy plant microbiome, mode of action, microbiome shift

## Abstract

Plant-associated microorganisms are involved in important functions related to growth, performance and health of their hosts. Understanding their modes of action is important for the design of promising microbial inoculants for sustainable agriculture. Plant-associated microorganisms are able to interact with their hosts and often exert specific functions toward potential pathogens; the underlying *in vitro* interactions are well studied. In contrast, *in situ* effects of inoculants, and especially their impact on the plant indigenous microbiome was mostly neglected so far. Recently, microbiome research has revolutionized our understanding of plants as coevolved holobionts but also of indigenous microbiome-inoculant interactions. Here we disentangle the effects of microbial inoculants on the indigenous plant microbiome and point out the following types of plant microbiome modulations: (i) transient microbiome shifts, (ii) stabilization or increase of microbial diversity, (iii) stabilization or increase of plant microbiome evenness, (iv) restoration of a dysbiosis/compensation or reduction of a pathogen-induced shift, (v) targeted shifts toward plant beneficial members of the indigenous microbiota, and (vi) suppression of potential pathogens. Therefore, we suggest microbiome modulations as novel and efficient mode of action for microbial inoculants that can also be mediated *via* the plant.

## Introduction

Plants are naturally associated with specific microorganisms, which fulfill important functions, e.g., nutrient, mineral and vitamin supply, and protection against biotic and abiotic stress (Vandenkoornhuyse et al., 2015; Sánchez-Cañizares et al., 2017; Bakker et al., 2020). Microbiome research has revolutionized our understanding of plant microbiome functioning, and it simultaneously opened new possibilities for applications toward sustainable agriculture (Berg et al., 2020). Advances in engineering of environmental microbiomes are predicted to replace toxic chemicals and fertilizers in agriculture in the future, and stimulate a more sustainable use of environmental resources, as well as improve our food processing (Massart et al., 2015). Currently, agricultural products based on microorganisms are one of the fastest growing sectors in agronomy with a Compound Annual Growth Rate (CAGR) of 15–18% and a predicted value of over $10 billion United States dollars by 2025 for the whole biocontrol sector (DunhamTrimmer, 2017).

Understanding their modes of action is important for the design of promising applications in the form of microbial inoculants for sustainable agriculture (Berg, 2009; Köhl et al., 2019). Microbial inoculants comprise bioprotectants, biopesticides, and biostimulants, as well as biofertilizers (Lugtenberg and Kamilova, 2009; DunhamTrimmer, 2017). However, these classifications, which are mostly based on to the underlying modes of action, are currently under discussion. Moreover, there are various country-specific as well as continent-specific definitions (ibma-global.org). From the scientific point of view, most of microbial inoculants influence the host plant by stimulating plant defense response, plant hormone production and nutrient uptake (Windisch et al., 2017). Thus, the categories listed above might be important for regulation purposes but are still poorly defined, because inoculants that improve plant growth might also enhance plant resistance toward pathogens. Here, we use the term microbial inoculant without further categorizing it. Many of the currently available inoculants are able to establish interactions by relying on several modes of action, and their interactions depend on environmental conditions (Köhl et al., 2019). Despite this fact, interaction with the indigenous plant microbiome are often not considered at all.

*In vitro* plant-microbe as well as pathogen-microbe interactions are well studied (reviewed by Whipps et al., 1988; Weller et al., 2002; Berg, 2009; Köhl et al., 2019), while interactions with microbial inoculants *in situ* are not yet fully understood. Here, three-way interactions with the plant, with pathogens and with the indigenous microbiome are possible to induce beneficial effects. In general, the competence of microbial inoculants to colonize plant habitats is essential for successful interactions (Lugtenberg and Kamilova, 2009). Distinct steps during the initiation of such interplay include recognition, adherence, invasion (only endophytes and pathogens), colonization and growth, and several specific strategies to establish interactions (Hardoim et al., 2015). Direct interactions of microbial inoculants with host plants include (i) provision of nutrients and minerals, (ii) balancing the hormonal status of plants, and (iii) priming and induction of resistance (Schenk et al., 2010; Pieterse et al., 2014). Moreover, the ability to suppress pathogens can be an important feature (Weller et al., 2002). Pathogen suppression can be based on (i) antibiosis: inhibition of microbial growth by bioactive substances, (ii) competition (e.g., for iron) and modification of microenvironments, (iii) interference with pathogenicity, and (iv) parasitism and lysis (Lugtenberg et al., 2002; Linares et al., 2006). Furthermore, a meta-analysis by Mawarda et al. (2020) illustrates the impact of microbial inoculants on the native soil community structure and functioning and highlights changes in root exudation as an important underlying mode of action. Here, we summarize the current knowledge related to the effects of microbial inoculants on the indigenous plant microbiome, and suggest this novel, so far mostly unexplored mode of action to be termed “microbiome modulation.”

## Current Knowledge Related to Plant Microbiome Interactions

Plant microbiomes are characteristic microbial communities in habitats that are well-defined by distinct physio-chemical properties, e.g., in the rhizoplane (the surface of the root), rhizosphere (the soil influenced by the root), phyllosphere (stem and leaves) and the endosphere (inner plant parts) (Philippot et al., 2013; Hardoim et al., 2015; Remus-Emsermann and Schlechter, 2018). The plant and its associated microbiome can also be regarded as a meta-organism the so-called “holobiont” (Vandenkoornhuyse et al., 2015). Plant microbiome assembly starts with seeds, which are an important reservoir of microorganisms (Berg and Raaijmakers, 2018). The current model of plant microbiome assembly indicates that both the seed and soil microbiome are able to colonize the plant seedling, which allows to maintain microbial diversity and function but also facilitates adaptation to the local environment (Bergna et al., 2018). The phyllosphere microbiome is also recruited from airborne dust or irrigation water (Vorholt, 2012). Moreover, recent experimental evidences on the seed’s role in vertical transmission across plant generations showed high spatial partitioning of the fungal and bacterial community, within both seed and seedling, indicating inheritance, niche differentiation, and divergent transmission routes for the establishment of root and phyllosphere communities (Rezki et al., 2016; Wassermann et al., 2019; Abdelfattah et al., 2021). Distinct microbes from the surrounding environment can colonize the plants surface and tissues, especially in the rhizosphere, which is the soil influenced by the plant root and as such the interface to the soil. Here, the plant species, its specific rhizo-deposits and exudate blend constitute the main factors shaping the microbiome assembled in the vicinity of the roots (Neumann et al., 2014; Schreiter et al., 2014a). However, the plant growth developmental stage, soil properties and diverse agricultural management practices also strongly shape the rhizosphere microbiome and can influence plant microbiome assembly (Paul Chowdhury et al., 2019; Smalla pers. communication).

The plant-associated indigenous microbiome contributes with multiple functions to the holobiont, including (i) germination and growth, (ii) nutrient supply, (iii) resistance against biotic and abiotic stress factors, (iv) production of bioactive metabolites, (v) plant development and flowering, and (vi) attraction of pollinators and of predators and parasites of herbivores (Pieterse et al., 2014; Wagner et al., 2014; Berg et al., 2016; Etemadi et al., 2018). To fulfill these functions, plant-associated microorganisms are embedded in complex networks with microorganisms of the same species as well as with different species, genera, families and domains of life (Wassermann et al., 2019). The cooperation between plants and millions of microorganisms as well as the intra-microbiome interplay requires an intense communication (Venturi and Keel, 2016). A high number of specific genes encoding quorum sensing components and other signaling molecules were found in plant-associated microbial metagenomes (Bragina et al., 2014); however, the mechanism by which microorganisms interact as a community to confer beneficial traits to plants is still poorly understood. Quorum sensing is an essential communication factor, while a high number of volatile organic compounds are responsible for “microbial small talk,” but they can also act as long-distance messengers for interactions with the plant host (Schmidt et al., 2016). A review by Rosier et al. (2016) highlights the importance of chemical signaling, and biochemical and genetic events which determine the efficacy of benign microbes in promoting the development of beneficial traits in plants (Rosier et al., 2016).

## What Is a Healthy Plant Microbiome?

Although this question is of fundamental importance, we still cannot answer it entirely. Healthy plant microbiomes are characterized by a high microbial diversity and high evenness within the plant-associated indigenous microbial communities (Berg et al., 2017; Trivedi et al., 2020). However, all microhabitats harbor common and specifically adapted microbial communities; the diversity and evenness is generally lower in the rhizoplane (surface of the roots) and in the rhizosphere of crops compared to bulk soils, because only distinct microbes are attracted *via* chemical gradients to the root where they proliferate. Interestingly, all healthy plant microbiomes naturally also contain potential pathogens in seeds as well as in the rhizosphere (Berg et al., 2005; Wassermann et al., 2019). Manzotti et al. (2020) showed that after isolation and re-inoculation, they could exert their pathogenicity on the same host plant (Manzotti et al., 2020). This is an interesting observation underlying the conclusion that the balance and evenness within the plant microbiome is crucial for plant health. Each healthy plant microbiome is characterized by key stone species within the core microbiome and a dense network of positive interactions among the constituents (Berg et al., 2020). Following this perspective, the beneficial interplay of the host and its microbiome is essential for maintaining the health of the holobiont, while diseases are often correlated with a microbial dysbiosis or reduced diversity.

In the context of dysbiosis, the “pathobiome” concept, which integrates pathogenic agents within biotic environments, was established and applied to multiple pathosystems (Vayssier-Taussat et al., 2014). Analyses of pathobiomes often revealed that multiple pathogens and their followers (opportunistic microbes causing secondary infections) might be involved in a severe dysbiosis. Another interesting interpretation of microbial-host interactions is a so-called “Anna Karenina principle” (Zaneveld et al., 2017). It states that, paralleling Leo Tolstoy’s dictum that “all happy families look alike; each unhappy family is unhappy in its own way,” dysbiotic individuals vary more in microbial community composition than healthy individuals. For instance, infestations by *Bactrocera oleae* in olives was found to alter the natural fungal community into a dysbiotic state that was found to be unique in every geographical location (Abdelfattah et al., 2018). Dysbiosis was also observed in the rhizoplane of apple (“M26”) roots exposed to apple replant diseased soil. A strong local plant defense response resulted in phytoalexin accumulation, severe disease symptoms of the roots and in a dysbiosis (reduction of the alpha diversity) of the bacterial community (Balbín-Suárez et al., 2020). These results also suggest that microbial diversity is a key factor in preventing diseases in plants (Berg et al., 2017). Despite numerous studies, defining a “healthy microbiota,” the conception of borders between eubiosis and dysbiosis still remains a major challenge for the future of plant microbiome research.

## Interactions of Microbial Inoculants With the Plant and Its Microbiome

### Establishment of Microbial Inoculants

The establishment of microbial inoculants was studied for a long time, under different perspectives and by applying different methods. The used methodology has a crucial impact on results; highly sensitive methods, e.g., specific qPCR can improve studying colonization rates compared to e.g., cultivation-dependent methods (Verginer et al., 2010). However, the majority of studies revealed only transient establishment or low abundance of the microbial inoculants during plant growth (Scherwinski et al., 2008; Schreiter et al., 2014b; Eltlbany et al., 2019). In order to understand establishment efficiency, it is important to consider rhizosphere and rhizoplane colonization patterns of the inoculant (Götz et al., 2006; Elsayed et al., 2020). A successful establishment depends on strain traits (Adesina et al., 2009), application mode (Götz et al., 2006; Schmidt et al., 2012), and on the structure of the target microbiome (van Elsas et al., 2012). Schreiter et al. (2014b, 2018) showed that the competence of *Pseudomonas* sp. RU47 to colonize field-grown lettuce and potato roots was not influenced by the soil type (Schreiter et al., 2014b, 2018). High microbial diversity was shown to correlate with low colonization rates of typically non-plant/soil microbiome invaders as observed for *E. coli* (van Elsas et al., 2012) and *Salmonella* (Schierstaedt et al., 2020); the indigenous rhizosphere microbiome acts as a barrier or even protection shield of the holobiont against external intruders. Therefore, Adam et al. (2016) suggested the use of minor disturbance, e.g., selectively emptying niches (*via* introduction of bacterial predators, targeted antibiotics or enzymes), combined with timely application of microbial inoculants with or without helper strains to improve their establishment (Adam et al., 2016).

### Microbiome Modulation by Microbial Inoculants

The effect of microbial inoculants on the indigenous microbiome of plants has been studied now for more than one decade. Pioneering studies based on microbial fingerprinting techniques already revealed substantial shifts within microbial communities, e.g., caused by *Stenotrophomonas* or *Pseudomonas* treatments (Götz et al., 2006; Adesina et al., 2009; Schmidt et al., 2012). Application of next-generation sequencing techniques in the last years allowed disentangling these shifts in more detail. Exemplary interactions of microbial inoculants with the plant microbiome, including plant growth promoting bacteria, nitrogen fixing inocula, arbuscular mycorrhizal fungi as well as bacterial and fungal strains with antagonistic activity toward phytopathogens, are listed in Table 1.

**TABLE 1:** Microbial modulators and their influence on the plant microbiome.

Microbial inoculant	Pathosystem, pathogen, plant	Microbiome response	Microbiome modulation concept	References
*Sinorhizobium meliloti* L33	*Medicago sativa*	Microbiome shift: Decrease of γ-proteobacteria and increased of α-proteobacteria	E, F	Schwieger and Tebbe, 2000
*Serratia plymuthica* 3Re4-18 *Pseudomonas trivialis* 3Re2-7 *Pseudomonas fluorescens* L13-6-12	*Rhizoctonia solani* AG1-IB—lettuce	Short-time microbiome shift	A	Scherwinski et al., 2008
*Pseudomonas jessenii* RU47	*Rhizoctonia solani* AG1-IB—lettuce	Stabilization of microbial diversity Reduction of the pathogen-induced shift	B, C, and D	Adesina et al., 2009
*Paenibacillus sp.* E119 *Methylobacterium mesophilicum* SR1.6/6	Potato	Shift of Alphaproteobacterial and *Paenibacillus* communities	E	Andreote et al., 2010
*Stenotrophomonas rhizophila* SPA-P69	Cotton, tomato, and sweet pepper	Depletion of potential (minor) pathogens	F	Schmidt et al., 2012
*Serratia plymuthica* 3RE4-18 *Trichoderma viride* GB7 Consortium of both strains	*Rhizoctonia solani* AG1-IB—lettuce	Stabilization of microbial diversity	B, C	Grosch et al., 2012
*Chryseobacterium indologenes* ISE14 *Pseudomonas corrugata* CCR80	*Phytophthora capsici*—pepper	Increase of microbial diversity Targeted shifts toward potential beneficial phyla (*Pseudomonads* and *Actinomycetes)*	B, E	Sang and Kim, 2012
*Bacillus subtilis* B579	*Fusarium oxysporum* f. sp. *cucumerinum*—cucumber	Increase of microbial diversity, especially during flowering	B	Chen et al., 2013
*Pseudomonas fluorescens* 2P24 *Pseudomonas fluorescens* CPF10	Cucumber	Targeted shifts toward potential beneficial phyla (*Bacillus* spp.)	E	Yin et al., 2013
*Bacillus amyloliquefaciens* FZB42	*Rhizoctonia solani* AG1-IB—lettuce	Short-time microbiome shift	A	Chowdhury et al., 2013; Kröber et al., 2014
*Streptomyces subrutilus* Wbn2-11 *Bacillus subtilis* Co1-6 *Paenibacillus polymyxa* Mc5Re-14 *Pseudomonas fluorescens* L13-6-12 *Stenotrophomonas rhizophila* SPA-P69 *Serratia plymuthica* 3Re4-18	Chamomile	Treatment-specific microbiome shift, e.g. *Stenotrophomonas:* increase *Verrucomicrobia; Bacillus:* increase *Acidobacteria*	E	Schmidt et al., 2014
*Bacillus amyloliquefaciens* FZB42	*Rhizoctonia solani* AG1-IB—lettuce	Increase of microbial diversity Targeted shifts toward potential beneficial phyla (*Acinetobacter* spp. and *Alkanindiges* spp.)	B, E	Erlacher et al., 2014
*Ochrobactrum sp.* NW-3	Cucumber	Increase of microbial diversity	B	Song et al., 2015
*Bacillus amyloliquefaciens* NJN-6	Fusarium wilt- banana	Increase of bacterial diversity Microbiome shift: Abundance of *Acidobacteria, Firmicutes*, increased while abundance of *Fusarium* and fungi in general decreased	B, E, and F	Shen et al., 2015
*Stenotrophomonas acidaminiphila* BJ1	*Vicia faba*	Increase of microbial diversity	B	Zhang et al., 2017
Arbuscular mycorrhizal fungus *Rhizophagus intraradices*	Native shrub species	Microbiome shift: *Anaerolineaceae* family was an indicator of AMF-inoculated rhizospheres	A, E	Rodríguez-Caballero et al., 2017
*Trichoderma harzianum* CCTCC-RW0024	*Fusarium graminearum*—maize	Targeted shifts toward potential beneficial phyla (*Acidobacteria*) Depletion of potential pathogens (*Fusarium graminearum*)	D, E, and F	Saravanakumar et al., 2017
*Ensifer sp.* NYM3 *Acinetobacter sp.* P16 *Flavobacterium sp.* KYM3	Cucumber	Microbiome shift: Increase of *Gammaproteobacteria, Acidobacteria, Nitrospirae*, and *Armatimonadetes* Decrease of *Actinobacteria* and *Firmicutes* by microbial co-inoculations	E, F	Wang et al., 2018
*Bacillus cereus* AR156 *Bacillus subtilis* SM21 *Serratia sp.* XY21	*Phytophthora capsici*—pepper	Targeted shifts toward potential beneficial phyla (*Burkholderia spp., Comamonas spp., Ramlibacter spp.*)	E	Zhang et al., 2019
*Trichoderma harzianum* T-22 *Pseudomonas sp.* DSMZ 13134 *Bacillus amyloliquefaciens* FZB42 *Pseudomonas sp.* RU47	Tomato	Targeted shifts toward potential beneficial phyla (plant growth promoting bacteria)	E	Eltlbany et al., 2019
*Stenotrophomonas rhizophilia* SPA-P69	Maize	Targeted shifts toward potential beneficial phyla (plant growth promoting bacteria)	E	Kusstatscher et al., 2020
*Streptomyces pactum* Act12 *Streptomyces rochei* D74	Soil-borne pathogens—monkhood (*Aconitum carmichaelii*)	Strong microbiome shift Decrease of pathogens	A, F	Li et al., 2020
*Bacillus velezensis* B63 *Pseudomonas fluorescens* P142	*Ralstonia solanacearum* B3B- tomato	Targeted shifts toward potential beneficial phyla (*Actinobacteria, Verrucomicrobia)*	E	Elsayed et al., 2020
*Paenibacillus pasadenensis* R16 *Pseudomonas syringae* 260-02 *Bacillus amyloliquefaciens strain* CC2	*Rhizoctonia solani* AG1-IB/*Pythium ultimum*—lettuce	Strong microbiome shift Reduction of *Pythium ultimum* symptoms	A, D, and F	Passera et al., 2020
*Bacillus amyloliquefaciens* W19	*Fusarium oxysporum f. sp. cubense* (FOC) —banana	Targeted shifts toward potential beneficial phyla (*Pseudomonas* spp.)	E	Tao et al., 2020
*Brevibacterium frigoritolerans* HRS1 *Bacillus niacini* HRS2 *Solibacillus silvestris* HRS3 *Bacillus luciferensis* HRS4	*R. solanacearum*—tomato	Compensation or reduction of the pathogen-induced shift Consortium of functional guilds of healthy plants restoring the dysbiosis	D	Lee et al., 2021
*Bacillus amyloliquefaciens strain* GB03	*R. solanacearum*—tomato	Microbiome shift which synchronized neighboring plants rhizosphere microbiome (VOC mediated)	A, E	Kong et al., 2020

*Microbiome modulation concepts: transient microbiome shifts (A), stabilization or increase of microbial diversity (B), stabilization or increase of plant microbiome evenness (C), restoration of a dysbiosis/compensation or reduction of a pathogen-induced shift (D), targeted shifts toward plant beneficial members of the indigenous microbiota (E), and suppression of potential pathogens (F) are indicated.*

Schwieger and Tebbe (2000) analyzed the effect of field inoculation with *Sinorhizobium meliloti* L33 on bacterial communities in rhizospheres of *Medicago sativa;* they were the first describing microbiome shifts. Transient microbiome shifts were also reported as response to diverse microbial inoculants in lettuce (Scherwinski et al., 2008). Moreover, a stabilization (increase of resilience to biotic and abiotic stresses) of the microbiome was often described; a specific case during the flowering period was observed after an inoculation of cucumber with *Bacillus subtilis* B579. In this study, *B. subtilis* increased the microbial diversity transiently by promoting the secretion of root exudates during flowering of the host plant. This resulted in an enhanced protection against the pathogen *Fusarium oxysporum* f. sp. *cucumeris*. Disease suppression of Fusarium wilt caused by *Fusarium oxysporum* f. sp. *cubense* (FOC) was observed after inoculation with *Bacillus amyloliquefaciens*, which indirectly supported the growth of the indigenous *Pseudomonas* population (Tao et al., 2020). Interestingly, the response of the indigenous rhizosphere microbiome of field-grown lettuce to the inoculant *Pseudomonas* sp. RU47 was soil type dependent as revealed by amplicon sequencing (Schreiter et al., 2014a). However, it remains to be shown whether the microbiome shifts occur at the sites were inoculants are colonizing or a distant site. The latter would indicate that microbiome modulations are not caused by direct interaction but by changes in the composition and amounts of exudates. Recently, Windisch et al. (2017) showed that both, pathogen inoculation as well as treatments with the bacterial inoculants *Pseudomonas* sp. RU47 or *Serratia plymuthica* 3Re-4-18 resulted in increased concentrations of the antifungal compounds benzoic and lauric acid in a soil type-dependent manner. Bottom rot of lettuce caused by *Rhizoctonia solani* I-IB was least severe in loess loam soil and correlated with the highest concentrations of both antifungal compounds. Obviously, an important factor in the complex interaction below ground is the plant and its chemical response to inoculants, pathogens or the indigenous soil microbiome. A similar holobiont-level mode of action protecting cucumber was shown to rely on the induction of a microbiome shift by *Pseudomonas fluorescens* 2P24 or CPF10 that modify the bacterial community composition toward *Bacillus* spp. (Yin et al., 2013). An additional mode of action was observed with *Pseudomonas* sp. RU47, which stabilized the plant’s microbiome. The bioprotectant counteracted diversity losses that are observed in combination of *Rhizoctonia solani* AG1-IB (Adesina et al., 2009). An analogous effect was observed in combination with *R. solani* AG1-IB following a co-inoculation of lettuce with *Serratia plymuthica* 3Re4-18 and *Trichoderma viride* GB7. The combination of these two strains enhanced the biocontrol efficacy and increased the evenness of the plant microbiome (Grosch et al., 2012). Similar effects were observed in tomato plants infected with *Ralstonia solanacearum* B3B through the inoculation of *Bacillus velezensis* B63 or *Pseudomonas fluorescens* P142. Here, a microbiome shift toward genera that comprise multiple strains with plant growth promoting activity such as *Arthrobacter* and *Gaiella (Actinobacteria*) or *Ochrobactrum* (*Alphaproteobacteria*) was observed (Elsayed et al., 2020). *Fusarium* stalk rot in maize caused by *Fusarium graminearum* can be controlled *via* application of *Trichoderma harzianum* CCTCC-RW0024, which resulted in an increase of plant growth promoting *Acidobacteria* (Saravanakumar et al., 2017). Furthermore, a significant increase in microbial activity was observed following the inoculation of pepper plants infected with *Phytophthora capsici* with *Pseudomonas corrugata* CCR80 or *Chryseobacterium indologenes* ISE14. Both biocontrol agents increased the microbial diversity and induced microbial shifts toward *Pseudomonas* and *Actinomycetes* that contain many strains that are well-known for their antagonistic potential (Sang and Kim, 2012). When a bacterial consortium consisting of the PGPR strains *Bacillus cereus* AR156, *Bacillus subtilis* SM21 and *Serratia sp*. XY21 was used for the inoculation of sweet pepper infected with *Phytophthora capsici*, the disease symptoms were significantly reduced. The consortium significantly increased the abundance of the genera *Burkholderia, Comamonas*, and *Ramlibacter*. *Burkholderia* spp. are common antagonists of various plant pathogens, while *Comamonas* spp. commonly have antifungal effects and *Ramlibacter* spp. can potentially improve plant growth under adverse conditions (Zhang et al., 2019). The extent of interferences of microbial inoculants with the plant microbiome can also differ and is likely strain specific. While *Stenotrophomonas rhizophila* SPA P69 was only able to suppress minor fungal pathogens within the rhizosphere microbiome (Schmidt et al., 2012; Kusstatscher et al., 2020), *B. velezensis* FZB42 was observed to generally increase the microbial diversity in the rhizosphere as inferred from 16S rRNA amplicon analyses, which led to the identification of an advanced mode of action against *Rhizoctonia solani* AG1-IB (Erlacher et al., 2014). Chowdhury et al. (2013) reported similar results; they showed that FZB42 treatments resulted in a shift of the indigenous rhizosphere bacterial community although the inoculant was only temporarily present. Interestingly, Lee et al. (2021) were the first to demonstrate that dysbiosis of the protective Gram-positive bacterial community in diseased tomato plants promotes the incidence of disease. This underlines the importance of this novel mode of action. Moreover, the same group showed a plant mediated microbiome shift (*via* volatile organic compounds and root exudates) in neighboring plants to the one inoculated with *Bacillus amyloliquefaciens* GB03 (Kong et al., 2020).

**FIGURE 1:**
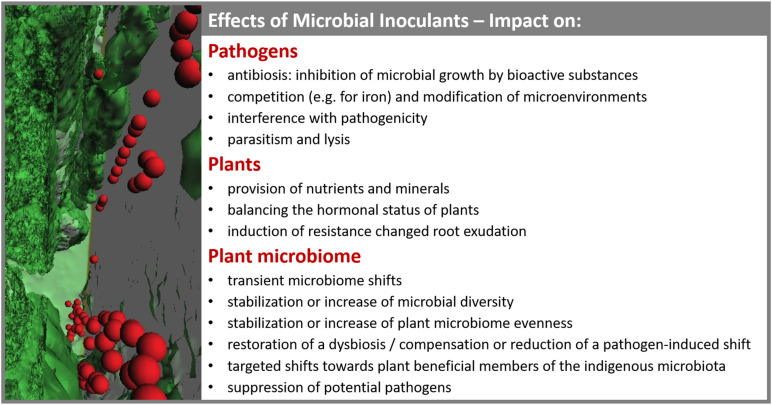
Summary of known effects elicited by microbial inoculants on pathogens, plants and the plant-associated microbiome. Various effects of inoculants on native plant microbiomes are connected to their modes of actions related to plant health and disease prevention. The included 3D reconstruction of a micrograph shows the colonization of a microbial inoculant in plant tissues.

In general, six different types of microbiome modulation were described in literature: (i) transient microbiome shifts, (ii) stabilization or increase of microbial diversity, (iii) stabilization or increase of microbiome evenness, (iv) restoration of a dysbiosis/compensation or reduction of a pathogen-induced shift, (v) targeted shifts toward potential beneficial phyla, and (vi) depletion of potential pathogens. Microbiome modulations are an important response to microbial inoculants, which should be considered in terms of the impact on plants and pathogens (Figure 1).

### Methods to Study Microbiome Modulation by Microbial Inoculants

The detectable inoculation impact on the plant microbiome depends on the time of sampling (days or weeks after inoculation) and more generally on the technical approach (Mawarda et al., 2020). Currently, the state of the art for analyzing plant or rhizosphere microbiome shifts in response to microbial inoculants are total community DNA based methods. Although these methods are prevalent, it is important to mention that a large number of publications is available that highlight the bias in DNA extraction and processing procedures for subsequent analysis of microbiomes (Albertsen et al., 2015). PCR-amplified 16S rRNA genes (bacteria, archaea) or ITS fragments amplified from total community DNA were analyzed by fingerprinting methods in the past and presently by amplicon sequencing. The latter method is suitable to unravel the taxonomic composition but also shifts in the relative abundance or diversity changes. So-called responders to the treatments (microbial inoculants)—taxa that significantly increased or decreased in relative abundance—can be determined by comparative analyses of amplicon sequence data from inoculated and non-inoculated treatments. To avoid studying relic DNA or such from dead cells, propidium monoazide (PMA) can be implemented. It can only penetrate membrane-damaged cells, where a photo-induced azide group covalently binds to DNA and effectively inhibits PCR amplification of DNA from dead cells of both Gram-negative and Gram-positive bacteria (Nocker et al., 2006). Short read sequences with typically 150–300 bp read length from Illumina high throughput sequencing should provide sufficient sequencing depth after quality filtering and data processing to obtain taxonomic composition on genus level. Even though the number of reads necessary to obtain full diversity in samples is impossible to define, beta diversity is usually covered with a lower sequencing depth, while higher read numbers are needed for alpha diversity and rare taxa (Gołȩbiewski and Tretyn, 2020). Compositional and microbiome shift observations are possible with this data, however, functional predictions based on 16S rRNA and ITS region data should be carefully interpreted. Presently, we mainly compare detected taxa to what is known from isolates of the particular species. However, it is also well known that plant-beneficial traits are typically strain-specific. Strains sharing identical 16S rRNA gene sequences might have diverse plant-beneficial traits. Thus, linking amplicon sequencing with plant-beneficial functions remains challenging. Conducting cultivation-based characterization in parallel has the advantage that strain-specific traits ranging from rhizosphere competence, *in vitro* antagonistic activity, production of extracellular enzymes, siderophores, indol acetic can be determined. Even though, it must be mentioned that *in vitro* effects may not always represent *in vivo* effects of a certain strain. Moreover, whole genome sequencing allows to precisely determine the taxonomy by multi-locus sequencing and comparative genomics (Loper et al., 2012; Kuzmanović et al., 2019). Furthermore, interaction studies with the plant can be performed and the effects on plant hormones, secondary metabolites, root growth mineral uptake, and the indigenous microbiome can be assessed. Metagenome sequencing and single cell genomics (based on Raman or other high throughput isolation and screening techniques) have enormous potential to disentangle inoculant-induced microbiome shifts with cultivation-independent methods (Jansson and Baker, 2016; Song et al., 2017; Zhang et al., 2021). Moreover, evaluating inoculants from a functional perspective using a broad range of omic approaches is important to assess their impact on ecosystem functioning (Mawarda et al., 2020). Most critical is the sensitivity of the cultivation independent methods that will likely detect shifts in the dominant members of the plant microbiome (Blau et al., 2018). Microbiome research is strongly driven by methodological advances and, until now, there is no perfect and universal method. It can be expected that in the future an improved toolbox of technologies will reduce bias resulting from each individual technology and result in a more complete view on the biological system as a whole (Berg et al., 2020).

### Assessment and Integration of Microbiome Modulations Into Mode of Action Patterns

Interestingly, no study so far described an enrichment of potential pathogens due to inoculant application, neither for plants nor for humans. In contrast, many studies described an enrichment of well-known plant beneficial bacteria, e.g., *Pseudomonas* (Sang and Kim, 2012; Tao et al., 2020), *Bacillus* (Yin et al., 2013), *Acidobacteria* (Saravanakumar et al., 2017; Wang et al., 2018), *Burkholderia, Comamonas, Ramlibacter* (Zhang et al., 2019), and *Verrucomicrobia* (Schmidt et al., 2014; Eltlbany et al., 2019). In this context, an interesting study was published by Lee et al. (2021). They identified a higher relative abundance of *Actinobacteria* and *Firmicutes* in healthy tomato plants. Representative isolates of the health indicators (*Brevibacterium frigoritolerans* HRS1, *Bacillus niacini* HRS2, *Solibacillus silvestris* HRS3, and *Bacillus luciferensis* HRS4) were able to induce an immune activation and extended plant protection against *R. solanacearum.* In addition, several studies described the depletion of potential pathogens due to inoculant application (Schmidt et al., 2012; Saravanakumar et al., 2017; Elsayed et al., 2020). These studies provide indications that depletion of pathogens might be an accompanying effect to the enrichment of plant-beneficial strains, which together constitutes the observed microbiome shifts. Wang et al. (2018) analyzed different consortia and showed that the microbiome is specifically modulated by distinct microbial strains. Gu et al. (2020) reported that growth inhibitory siderophores secreted by microbial inoculants or members of the indigenous rhizosphere microbiome play an important role in suppressing the bacterial phytopathogen *R. solanacearum* and protecting plant health. They proposed a mechanistic link between microbiome competition for iron and plant protection (Gu et al., 2020). Such observations require further assessments in order to better understand the underlying molecular modes of action. The current knowledge base indicates that microbiome modulations are part of the interaction of microbial inoculants with plant hosts. Together with the ability to suppress pathogens, induce resistance, provide nutrients and minerals, and change/balance the hormonal status of plants, the interaction of microbial inoculants with the indigenous plant microbiome is important to consider. Moreover, secondary effects induced by modulating the native microbiome are possible, e.g., recruitment of other beneficial microbes.

## Discussion

**FIGURE 2:**
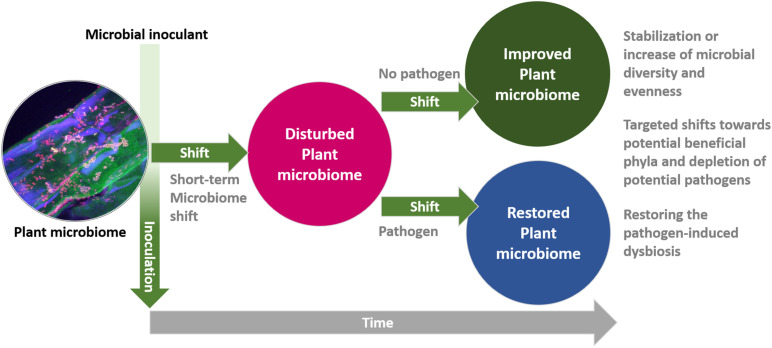
A model for plant microbiome responses to microbial inoculants. Inoculation of microbial inoculants induces short-term shifts and improves or restores a healthy plant microbial community on a long term.

Altogether, we have identified and described six different modulations types of the plant microbiome by microbial inoculants. According to available literature, the extent of modulation depends on the sampling time following the application. Directly after the application, strong, transient microbiome shifts are commonly observed. A few weeks after the application, shifts are often not evident anymore; however, at this point a stabilization effect or even an increase in microbial diversity and evenness was often described. This observation is in line with the intermediate disturbance hypothesis in macro-ecology, which suggests that local species diversity is maximized when ecological disturbance is neither too rare nor too frequent (Wilkinson, 1999). On the other hand, if the microbial ecosystem is already substantially disturbed, which is reflected by a severe dysbiosis, distinct microbial inoculants are able to restore the dysbiosis by compensation or a reduction of pathogen-induced microbiome shifts. Considering microbiome composition, all studies that are available so far describe shifts enriching potentially beneficial microbes while simultaneously decreasing potential pathogens. A summary of previous results was integrated in a model for plant microbiome responses to microbial inoculants (Figure 2).

Microbial inoculants are only one out of many different possibilities to manage microbiomes. While microbial inoculants are well-defined, formulated single or multi species preparations, highly diverse microbiome transplants from other environments or bio-active metabolites can also be used to directly manage microbiomes. In addition, by changing the environmental conditions, the structure and function of microbiomes can be shifted (Berg and Smalla, 2009). In general, various treatments were shown to induce similar patterns and microbiome shifts (Cernava et al., 2019; Guimarães et al., 2020). Surprisingly, breeding was shown to cause similar, long-term microbiome shifts as well (Pérez-Jaramillo et al., 2018). In the last centuries, plant breeding was directed to high yields and to resistance toward pathogens; high yield cultivars enriched plant growth promoting microorganisms (Pérez-Jaramillo et al., 2018), while resistant cultivars enrich microorganisms antagonistic toward pathogens (Adam et al., 2018; Mendes et al., 2019; Wolfgang et al., 2020). For example, *Rhizoctonia*-tolerant cultivars of sugar beet, mainly mediated by the Fort Collins Resistance (USDA), resulted in an enrichment of bioactive *Pseudomonas* strains in the rhizosphere. Strains of *Pseudomonas* are also able to induce the same resistance under experimental settings (Wolfgang et al., 2020). Only recently two independent studies have shown that microorganisms can shape their host phenotypes by evoking resistance traits that are undistinguishable from innate plant immunity: in sugar beets two members of the native endorhiza microbiota (*Chitinophaga* and *Flavobacterium)* were shown to confer resistance to the host plant against *Rhizoctonia solani* (Carrión et al., 2019) and in rice a seed-endophytic *Sphingomonas melonis* strain was shown to confer disease resistance against *Burkholderia plantarii* that causes seedling blight (Matsumoto et al., 2021). The available evidence for such functions strongly suggests integrating microbiome research into breeding and plant protection strategies. Management can also induce microbiome shifts; for example, biofumigation-modulated soil microbiomes in response to organic amendments and was proposed to be a major mode of action. The effects of biofumigation on apple plant growth were site-dependent and might result from suppression of soil-borne pathogens and changes in soil microbial community compositions and activity through the additional nutrients from the incorporated biomass (Yim et al., 2017). Thus, combining the activation of indigenous beneficial bacteria in the soil and microbial inoculants through suitable organic amendments seems a promising approach for microbiome modulation and enhancing plant growth. However, we have to take into account that also pathogens evolved to manipulate host microbiomes to their advantage by using effector proteins (Snelders et al., 2020).

Direct microbiome modulation by microbial inoculants or such that are mediated by the host plant provide important options for sustainable agriculture and circular bio-economy. The application of microbial inoculants to replace chemical pesticides and to use waste materials (especially agricultural residue) for their production is an important step in counteracting major problems of the future such as climate change, biodiversity loss and changes in biogeochemical cycles. It is highly interesting that similar types of microbiome modulations were detected in plants, animals and humans (Frerejacques et al., 2020; Ojima et al., 2020). The restoration of reduced diversity of the microbiome by administration of specific microorganisms was observed in animals as well as humans. This cross-kingdom similarity was already described for microbiome functioning (Mendes and Raaijmakers, 2015) and control (Berg et al., 2015). Due to their importance, microbiome shifts definitively require a better understanding of the underlying communication and interaction mechanisms within the microbiome. Further experimental evidence, utilizing gnotobiotic plant trials as well as field conditions, is needed to mechanistically explore the underlying mechanisms. Especially the role of the host in the microbiome modulation requires a better understanding, along with the molecular pathways involved in the host response. Multi-omics approaches can help to understand the interaction of microbial inoculants with all possible partners including the host, the microbiome and their interplay (Jansson and Baker, 2016). At least it is proposed to re-think current categories of microbial inoculants and their regulation to open new possibilities for applications toward sustainable agriculture.

## Author Contributions

GB drafted and refined the manuscript. All authors critically read the article, contributed to the writing, and approved it for publication.

## Conflict of Interest

The authors declare that the research was conducted in the absence of any commercial or financial relationships that could be construed as a potential conflict of interest.
